# The diagnosis of pulmonary embolism without contrastis not always Challenging: be aware of hyperdense lumen sign

**DOI:** 10.11604/pamj.2018.30.279.16283

**Published:** 2018-08-17

**Authors:** Eylem Kuday Kaykisiz, Erden Erol Unluer, Utku Eser

**Affiliations:** 1 Department of Emergency Medicine, Bitlis State Hospital, Bitlis, Turkey; 2 Department of Emergency Medicine, Usak University Faculty of Medicine, Usak, Turkey; 3 Department of Family Medicine, Usak University Faculty of Medicine, Usak, Turkey

**Keywords:** Pulmonary embolism, CTPA, unenhanced thorax CT

## Abstract

Acute pulmonary embolism (PE) diagnosis is a challenging task, despite the advanced diagnostic methods for both clinicians and radiologists. Awareness of the “hyperdense lumen sign” in patients obtained un-enhanced computarized tomography (CT) of chest mayhelp to establish an acute PE diagnosis, especially in clinically non suspected PE patients. A 78-year-old woman was brought to our emergency department (ED) with an aphasia complaint. The patient's dizziness improved in ED. Neurological examination returned to base line status but sinus tachycardia and low saturation value on room air were continuing. Un-enhanced CT of the chest demonstrates hyperdense material within the right main pulmonary artery. Contrast-enhanced CTPA demonstrated hypodense filling defect within the rigth main pulmonary artery consistent with PE. Independent of the patient's complaint, the measurement of all vital signs is important especially in elderly patients. Emergency physicians have to be aware of that the “hyperdense lumen sign” may point out PE and should be prevented from delayed recognition.

## Introduction

It may sometimes be difficult to diagnose in emergency room because of that elderly patients cannot express themselves adequately and symptoms in this age group do not match exactly with the disease's characteristics. Independent of the patient's complaint, the measurement of all vital signs is important especially in elderly patients at this point. Acute pulmonary embolism (PE) diagnosis is a challenging task, despite the advanced diagnostic methods for both clinicians and radiologists. The most important step in the diagnosis of the disease is clinical suspicion. The most reliable diagnostic method is contrast-enhanced CT pulmonary angiography (CTPA) in which intraluminal filling defect sare seen after IV contrast agent administration [1]. Awareness of the “hyperdense lumen sign” in patients obtained unenhanced computarized tomography (CT) of chest with various cardiopulmonary symptoms may help to establish an acute PE diagnosis, especially in clinically non-suspected PE patients [2]. We report a 78-year-old female patient who was admitted to emergency service due to dysarthria and obtained an un-enhanced CT of chest because of her suffered to low saturation and diagnosed with acute PE by a waring of hyperdenselumen sign. Our aim was to raise awareness that the “hyperdense lumen sign” might help in the diagnosis of acute PE in patients with undifferentiated dyspnea.

## Patient and observation

**Figure 1:**
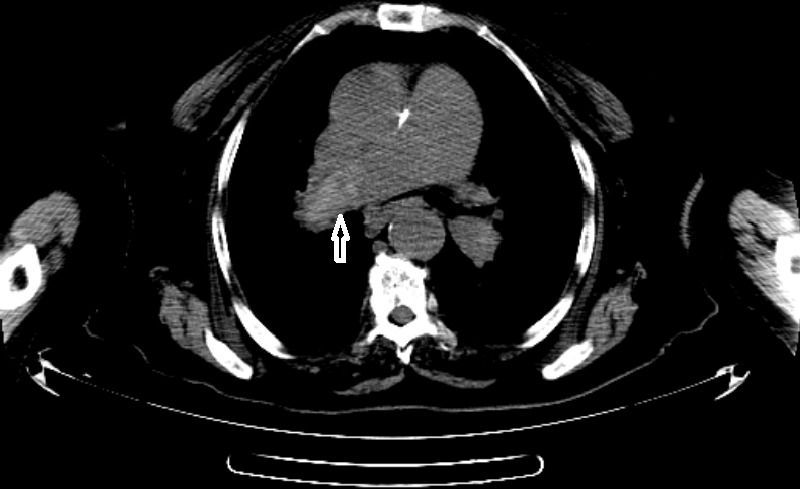
Unenhanced CT of the chest demonstrates hyperdense material within the right main pulmonary artery

**Figure 2:**
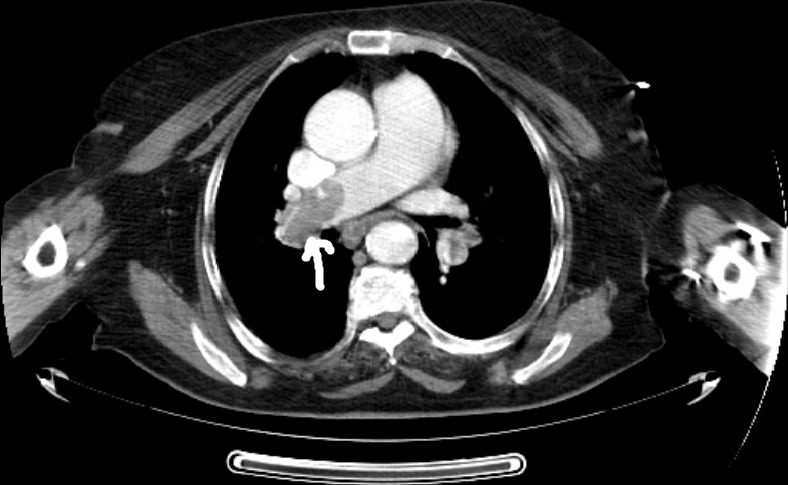
Pulmonary CT angiography with contrast demonstrates hypodense filling defect within the rigt main pulmonary artery consistent with pulmonary embolism

A 78-year-old woman was brought to our emergency department by her relatives with an aphasia complaint that had been intermittent for the last 6 hours. The patient's past medical history was unremarkable except hypertension. On original presentation, she was sub febrile with 37,8°C, with a blood pressure of 160/80mmHg, pulse of 105 beats/min, respiratory rate of 30 beats/min, and oxygen saturation of 88% on room air. On her detailed examination, she was well-oriented and cooperated, exhibiting no signs of cranial nerve function disorders, such as facial palsy or dysphagia except disarthri. Her motor strength was normal bilaterally throughout her upper and lower extremities. Distal pulses and sensations were intact in all extremities. No pathological reflexes were found bilaterally. The DTRs were normoactive in all extremities. Her sensory perceptions of touch, vibration and joint position were normal. Cerebellar function tests were negative and there were no signs of ataxia. Her electro cardiogram was noted as sinus tachycardia and right branch block. Laboratory values, including complete blood count, biochemistry and full urinanalysis, were within normal ranges except leukocytosis (WBC: 12.400/mm^3^) with neutrophil dominance. Arteriel blood gase analysis revealed that the patient was hypoxic but not hypocarbic (pH 7.41, Paco2 37 mm Hg and Pao2 68 mm Hg, saturation %88 on room air). Brain computerized tomography without contrast revealed chronic atrophic changes. Diffusion-weighted magnetic resonance imaging of the patient revealed high intensity signals adjacent to left lateral ventricle without apparent reduced diffusion coefficient in the same location so that this view was thought to be meaningful in terms of sub acute infarction. The patient's dizziness improved in the emergency room. Neurological examination returned to baseline status but sinus tachycardia in her electrocardiogram and low saturation value on room air were continuing. Obtained post ero-anterior pulmonary radiography was unremarkable. An un enhanced CT of chest was planned to patient with a preliminary diagnosis of pneumonia. Un enhanced CT of the chest demonstrate shy perdense material within the right main pulmonary artery (Figure 1). Also the main pulmonary artey and right pulmonary artery were enlarged that is indicative of pulmonary hypertension. Contrast-enhanced CTPA was planned because of seen hyperdense lumen sign and demonstrated hypodense filling defect within the rigth main pulmonary artery consistent with PE (Figure 2). Thrombolytic therapy was not planned because of the patient had sub acute infarct at the same time. The patient who was started anti thrombotic treatment was hospitalized by a pulmonogist.

## Discussion

Many patients in emergency services are suffering from un enhanced thorax CT for distinct reasons. These include; pneumonia, emphysema, interstitial pulmonary disease, bronchiectasis. In addition, patients with non-specific cardiopulmonary symptoms without contrast due to impaired renal function and contrast allergy are also suffering from un enhanced CT. Recent publications have shown that hyperdensity in the pulmonary artery in the un enhanced CT scan will indicate pulmonary embolism [3-6]. This was first described by Gotway MB et al in year 2000 [7]. However, the number of cases in the literature still does not pass the fingers of a hand [8]. False positivity rate is very low, but false hyperdense luminal imaging may occur due to artefacts around the pulmonary artery [2]. The hyperdense appearance of the acute thrombus is due to the increase in hemoglobin concentration in the clot due to decreased water content in the clot [8]. But this finding is rarely seen even if the patient has PE. Indirect findings such as pulmonary artery dilatation, pleural effusion, regional oligemia, and sub pleural pulmonary consolidation, which are not specific or sensitive, are well described [2]. However contrast enhanced CTPA is the most commonly used and reliable diagnostic method for PE, being aware of hyperdense lumen sign can help early detection of acute PE in patients with un suitable to contrast agent. In our case, the hyperdense lumen sign seen in un enhanced thorax CT obtained forun differentiated dyspnea, was headed us for acute PE and diagnosed in the early period after that the treatment started rapidly. However, confirmatory tests such as contrast-enhanced CTPA or ventilation perfusions cintigraphy are still recommended [5].

## Conclusion

Emergency physicians have to be aware of that the “hyperdense lumen sign” seen in un enhanced thorax CT obtained from the patients with various cardio pulmonary symptoms, may point out PE and should be prevented from delaying recognition with confirmatory tests in the early period.

## Competing interests

The authors declare no competing interest.
